# What Makes International Global Health University Partnerships Higher-Value? An Examination of Partnership Types and Activities Favoured at Four East African Universities

**DOI:** 10.29024/aogh.20

**Published:** 2018-04-30

**Authors:** Aaron N. Yarmoshuk, Anastasia Nkatha Guantai, Mughwira Mwangu, Donald C. Cole, Christina Zarowsky

**Affiliations:** 1 School of Public Health, University of the Western Cape, ZA; 2 University of Nairobi, KE; 3 Muhimbili University of Health and Allied Sciences, TZ; 4 Dalla Lana School of Public Health, University of Toronto, CA; 5 CR-CHUM/ESPUM, Université de Montréal, CA

## Abstract

**Background:**

There are many interuniversity global health partnerships with African universities. Representatives of these partnerships often claim partnership success in published works, yet critical, contextualized, and comparative assessments of international, cross-border partnerships are few.

**Objective:**

The objectives of this paper are to describe partnerships characterized as higher-value for building the capacity of four East African universities and identify why they are considered so by these universities.

**Methods:**

Forty-two senior representatives of four universities in East Africa described the value of their partnerships. A rating system was developed to classify the value of the 125 international partnerships they identified, as the perceived value of some partnerships varied significantly between representatives within the same university. An additional 88 respondents from the four universities and 59 respondents from 25 of the international partner universities provided further perspectives on the partnerships identified. All interviews were transcribed and analysed in relation to the classification and emergent themes.

**Findings:**

Thirty-one (25%) of the partnerships were perceived as higher-value, 41 (33%) medium-value, and 53 (42%) lower-value for building the capacity of the four focus universities. Thirteen (42%) of the higher-value partnerships were over 20 years old, while 8 (26%) were between 3 and 5 years old. New international partners were able to leapfrog some of the development phases of partnerships by coordinating with existing international partners and/or by building on the activities of or filling gaps in older partnerships. Higher-valued partnerships supported PhD obtainment, the development of new programmes and pedagogies, international trainee learning experiences, and infrastructure development. The financial and prestige value of partnerships were important but did not supersede other factors such as fit with strategic needs, the development of enduring results, dependability and reciprocity. Support of research or service delivery were also considered valuable but, unless education components were also included, the results were deemed unlikely to last.

**Conclusion:**

International partnerships prioritizing the needs of the focus university, supporting it in increasing its long-term capacity and best ensuring that capacity benefits realized favour the focus university are valued most. How best to achieve this so all partners still benefit sufficiently requires further exploration.

## Background

International, interuniversity partnerships, particularly North-South partnerships between universities in high-income countries (HICs) and sub-Saharan African (SSA) universities, have long been considered one means by which to increase the capacity of health professional programs (HPPs) of African universities [1,2,3,4,5,6]. The international partnership mix of SSA universities has become increasingly complex in recent years, with the development of partnerships between universities in low- and middle-income countries (LMICs) [i.e. South-South partnerships], North-South-South partnerships and consortium partnerships or networks [7,8].

The Sub-Saharan African Medical School Study suggested future research was needed on how to measure and improve partnerships with a view to improving efficacy and providing “evidence of success” [9, p. 95]. Mulvihill and Debas identified four “successful long-term academic partnerships” [10, p. 512], including one in which their university (University of San Francisco, USA) is involved and one between Indiana University in the USA and Moi University (MU) in Kenya. The Indiana-MU University partnership was cited as a positive example by Frenk et al. [6] as well. Crane [11] identified it as an example of a successful partnership. For Crane, the partnership is successful because the research and training outputs are reciprocal, and it is improving patient care at MU’s teaching hospital.

While asserting the potential value of universities globally in helping to address global health challenges, the Academy of Medical Sciences and Royal College of Physicians [8] noted that adequate evaluation of university partnerships is lacking. Analysis of partnerships themselves, and their limitations, is often lacking in detail. Mulvihill and Debas [10] cite only one or two references for each of their four examples of partnership success. All but one reference was authored by representatives of the partnerships and the source for the fourth one was in a report that included but one paragraph on the partnership [4].^1^

Of further concern is the interested nature of reports – Crane’s [11] only reference is a book written by an Indiana University representative [12]. After lamenting the low historic impact of many capacity building initiatives in low-income countries, Cancedda et al. [13] mention a partnership between the University of Oulu in Finland and the University of Namibia and Lurio University in Mozambique as innovative, citing only the University of Oulu’s web-site, before detailing four “innovative” projects that the authors “played a critical role” [p. 5] in developing and implementing. Having implementers writing about their own partnerships may be scientifically defensible, given the difficulties associated with an outsider obtaining a sufficient understanding of multi-year partnerships as complex interventions [14]. However, it does raise the question of *competing interest bias* [15] in scientific inquiry, even if authors identify their competing interests, especially in an era when the use of positive adjectives such as “innovative” in academic papers has increased significantly, likely in response to the pressure to publish and need to sell results [16].^2^

In a recent paper, we identified and mapped 129 international university partnerships from 23 countries that senior representatives^3^ of four East African universities – Moi University (MU), University of Nairobi (UoN), Kilimanjaro Christian Medical University College (KCMUCo), Muhimbili University of Health and Allied Sciences (MUHAS) – considered significant for strengthening their medicine, nursing and/or public health programs in education, research and/or service [17]. In addition to the usual descriptive characteristics (duration, partners involved, activities, etc.), how might we examine these through a more evaluative lens?

## Types of partnerships

Kernaghan [18], writing in the field of public sector management, classified partnerships into five broad categories or types, based on the degree to which power is shared within a partnership and, ultimately, the degree to which a partnership is empowering. In *collaborative* or “power sharing” partnerships, power is shared and resources are pooled. *Operational* partnerships are those that share work but not decision making. Power, or a sense of control, is retained by one partner. *Contributory* partnerships provide support (e.g. funding, resources), potentially increasing the ability of an organisation to perform a task. *Consultative* partnerships are interactions during which advice is provided from one partner to another. Kernaghan’s fifth type of partnership is *phoney* partnership, established with the intent to manipulate a partner and thus disempowering.

Although referring to the field of public sector management, Kernaghan’s model of five categories of partnership is a useful starting point for categorizing global health partnerships. In both fields, empowerment of at least one party is generally a goal. International university global health partnerships are often argued to be among unequals [19,20] and power is a concern when studying partnerships [21,22,23]. Moreover, the characteristics of Kernaghan’s top category of partnership, “collaborative”, are consistent with what is referred to in global health literature as “true partnership” [24], “real collaboration” [25], or “genuine collaboration” [26]. Collaborative partnerships are considered to be the gold-standard when it comes to two or more organizations working together in global health, a field many agree is concerned with addressing inequity within and/or between societies [27].

The objectives of this paper were to describe partnerships characterized as higher-value for building the capacity of four EA universities and identify why they are so considered by these universities.

## Methods

This study used a concurrent mixed methods design. Quantitative analysis was used to categorize the 125^4^ distinct partnerships identified and mapped previously into higher-, medium- and lower-value partnerships. Qualitative analysis was then used to determine the characteristics that contributed to the partnerships’ value, hence its dominant status [28] in this paper.

For the 129^5^ international university partnerships identified by 42 senior representatives of four SSA universities in our earlier work [17], we focused on the last two questions asked of the senior representatives: i) *How valuable (high, medium, low) was/is the partnership to your college or school (medicine, nursing and/or public health)?*; and, ii) *Please rank the partnerships in order of significance*.

In a 2^nd^ phase (November 2013 to July 2014), we conducted additional key informant interviews (KIIs) and focus-group discussions (FGDs) with lecturers, professors, staff and trainees from the four focus universities. Between 15 and 28 respondents participated per university (MU n = 28, UoN n = 23, KCMUCo n = 15, MUHAS n = 22, Total = 88). Trainees included medicine, nursing and public health students at various levels (Undergraduate, Masters, PhD, Residents, Fellows).^6^ At least one respondent from each of the universities’ health library was interviewed. At least one clinical medicine, basic science, nursing and public health lecturer and/or professor participated at all universities except for public health faculty at KCMUCo and basic science at UoN and MUHAS.

We used semi-structured interview guides for both the KIIs and FGDs to elicit representatives’ experiences within international partnerships and their perspectives of the benefits and challenges of the partnerships (See Appendix 4: Interview Guide for Phase 2 – FGDs with Senior Lecturers and Lecturers; Appendix 5: FGD Guide for Phase 2 – Students).

In a 3^rd^ phase (March 2014 to Nov. 2015), we conducted KIIs with 59 current or past representatives from 25 partner universities (African n = 3, European n = 9, North American n = 13) in nine countries (Canada n = 4, Egypt n = 1, Germany n = 1, Netherlands n = 2, South Africa n = 1, Sweden n = 5, Uganda n = 1, United Kingdom n = 1, United States n = 9) in-person or by phone/Skype. The vast majority of these KIs were currently or had been directly involved in the partnerships with one of the four focus universities in East Africa. Some of the respondents lived in Kenya or Tanzania so were interviewed there, with the remainder interviewed at their home institutions or at conferences. We adapted the earlier KI semi-structured interview guide for these international partners. We asked both general questions and questions specific to the partnerships in which they were involved (See: Appendix 6: Generic Interview Questions for International Partners – Phase 3). Additional information or clarification was sought from some KIs in follow-up interviews, via E-mail, telephone and/or SMS until the time this paper was submitted for publication.

Throughout the paper we have attempted to prevent attribution of specific comments to specific individuals. In those few circumstances where we felt this standard might not be met we contacted the individual(s) to determine if they wished to include a clarifying statement or rebuttal. In addition, we have not named specific international partners in partnerships not considered to be of higher-value, except when the partnership was viewed very positively but was mentioned by only one representative. We have named international partners in partnerships, who were considered to be higher-value, to illustrate perspectives on partnerships that do not appear to exist in the literature and to present limitations to “successful” partnerships missing in the literature.

### Ethics approvals

Ethics approval was obtained for the entire study (Phases 1, 2 and 3) from: the Senate Research Committee of the University of the Western Cape (13/5/15); Institutional Research and Ethics Committee Secretariat of Moi Teaching and Referral Hospital/Moi University School of Medicine; Ethics and Research Committee, Kenyatta National Hospital/University of Nairobi; and, National Institute for Medical Research in Tanzania. Research Clearance was received from the Tanzanian Commission for Science and Technology.

### Data management and analysis

From the Phase 1 data we added findings about the value of the partnership [17]. We calculated the value of each partnership by weighting the responses of the senior representatives. A response of *high* received a score of 5, a response of *medium*, 3 and a response of *low*, 1. Respondents who did not give a value for partners they identified were not included in the calculations for value, but their comments were included in the qualitative analysis. Partnerships that were mentioned by only one respondent were deducted 1 point so as not to inflate the number of *higher-* and *medium-value* partnerships, although their comments were included in the qualitative analysis. The scores for all respondents for the same partnerships were added and divided by the number of respondents who valued the partnerships to determine an average score. Partnerships receiving an average score greater than 4, and the top three most mentioned partners receiving no negative comments were classified as *higher-value* partners.^7^ Partnerships receiving an average score greater than 2 to 4 were classified as *medium-value*. Partnerships receiving an average score of 2 or less were classified as *lower-value*. We calculated the value of the three consortia identified by respondents at more than one of the universities using the same approach but included the responses of respondents from all the universities.

Content analysis was conducted [29] of all the interviews from Phase 1 to determine the characteristics associated with value in partnerships and to explore the perspectives on the dynamics of partnership development and producing value. Content analysis was also conducted of the interview from Phases 2 and 3 to add additional perspectives from representatives outside the decanal level of the focus universities and the international partners, respectively.

## Findings

### Partnership value

Overall, respondents were willing and able to classify partnership value: 31 (25%), were determined to be of higher-value, 41 (33%) medium-value, and 53 (42%) lower-value for building the capacity of the programmes (see: Table 1: Partnerships by Perceived Value for each Focus University).

**Table 1:** Partnerships by Perceived Value for each Focus University.

	MU	UoN	KCMUCo	MUHAS	Consortia Mentioned at more than 1 University

Higher-Valued	7	4	6	13	1
Medium-Valued	9	12	10	8	2
Lower-Valued	15	18	8	12	0
**TOTAL**	**31**	**34**	**24**	**33**	**3**

Nevertheless, four of the 42 (9.5%) KIs in Phase 1 when asked to state the value of each partnership as “high”, “medium” or “low” value found this request too difficult or too arbitrary to answer without having precise parameters. As one said, “I think it is very difficult because each one has had its own contribution, which is unique”. One KI, considered all the partnerships which they identified as “high” value while another stated, “No partnership can be low value”. When asked about the value of one partner’s contribution, one KI asked rhetorically, “Through one (research) project, is that helpful?” Some interviewees stated the “potential” of a partnership was medium or high value – e.g. “I’m looking at the others … and the tangible benefits” and then stated, “You cannot yet have tangible outcomes” in a new partnership. Only two representatives were willing to rank all of the partnerships they identified, although most KIs openly compared the approaches and results of partners when assigning value to each partnership.

### Where are higher-value partners from?

Twenty-six (26) of the higher-value partners were from the high-income countries (HICs), 13 from North America and 13 from Europe (see: Table 2: Higher-Value Partners Identified & Analysed).

**Table 2:** Higher-Value Partners Identified & Analysed.

Country	World Bank Income Group^8^	# of Higher-Value Partners	Total # of Partnerships Identified	% of All Partnerships Higher-Valued

USA	High	9	41	22%
Sweden	High	6	8	75%
Canada	High	4	6	67%
Germany	High	2	2	100%
Netherlands	High	2	4	50%
Consortium	Not applicable	2	10	20%
Denmark	High	1	2	50%
Kenya	Low	1	2	50%
Norway	High	1	7	14%
South Africa	Upper-Middle	1	8	13%
Uganda	Low	1	2	50%
UK	High	1	11	9%
Other Countries	Not applicable	0	22	0%
**TOTAL**		**31**	**125**	**25%**

Three (3) of the partners were from low-and-middle-income countries (LMICs), all within SSA – two were universities from low-income neighbouring countries^9^ and one from South Africa (Detailed findings of the higher-value partnerships of the four focus universities are provided in Appendix 1: Detailed findings of Higher-Valued Partners of each Focus University. The value of partnerships by country is provided in Appendix 2: Table of Partners by Country and Value of Partnership).

The two consortia determined to be higher-value included universities from Europe or the USA, although the majority of partners in each consortium was from SSA (see: Appendix 3: Table of Higher-Value Consortia – coordinating and partnering universities).

### Value by duration

**Figure 1:**
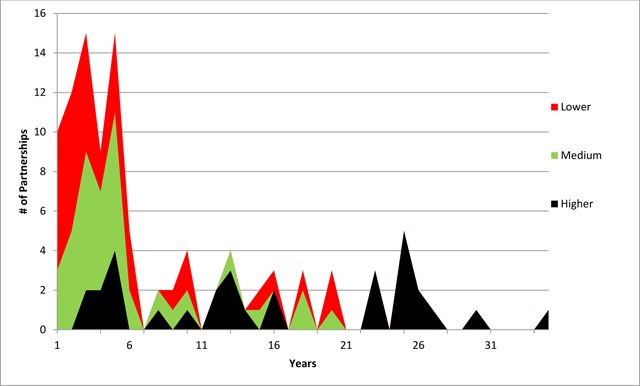
Area Graph of Partnerships by Value and Duration.

Thirteen (42%) of the 31 higher-value partnerships were older than 20 years, while 10 of 31 (32%) were 10 years or younger of which eight (26%) were between 3 and 5 years old. Over 70% of both lower- and medium-value partnerships were 5 years or younger, 26 of 37 (70.3%) and 27 of 37 (73%), respectively. Examples of lower-, medium- and higher-value partnerships can be found in every five-year duration range below 20 years (See Figure 1: Area Graph of Partnerships by Value and Duration).

### The role of funding

Funding levels of a partnership influenced the perceived value of the partnership to some degree. One representative began bluntly, “The higher the funding, the higher the impact for the university”, but then qualified the statement by adding, “there are partnerships (with smaller budgets) that are important for capacity building”. A second KI stated it’s not the dollar value “It’s what you get out of it”. A third KI noted: “If you don’t have funds the collaboration doesn’t survive”. Finally, a fourth KI responded: “It (i.e. money) is important but not the most important. The most important is really: what do you want to cooperate in … (and having) a common purpose”. This KI then concluded: “Of course, money becomes an issue. There needs to be a budget”. Lack of funding was often mentioned as challenge or weakness of a partnership. In many cases, KIs knew that a partnership was very active at their university but did not know how it was funded.

Salary compensation received by those participating in international partnerships was found to influence the perceived value to some extent. One KI stated, about a well-funded project, “If you don’t [provide] compensation for people when they are working on projects, they go to look somewhere else. The issue of salary compensation is [important] because of the low level of salaries paid by the government”. The same need for salary support was expressed by an international partner who had 7.5% of their salary covered by a project.

### Trainee-focused partnerships

In many partnerships involving only trainees, the international partner covered the cost of all beneficiary trainees (international and focus university) involved. Representatives often expressed the outputs in terms of simple ratios. Examples of the exchange ratio of trainees involved varied from approximately 1:1 (one international student to one focus university student exchanged) to 15:0 (15 international students to zero focus university students exchanged). Many of the senior SSA representatives did not expect 1 to 1 reciprocity. Others still valued unidirectional exchanges (e.g. HIC students travelling to East Africa, but not vice-versa) if the HIC students worked directly with their students; for example, on research projects. In addition to conducting research the interaction with international students was considered valuable by senior representatives. One partnership had international students travel to a focus university to be taught by its faculty. This was considered valuable for the opportunity to lecture another type of student and for the additional income faculty earned.

### Heterogeneity in perceptions of value

Nursing and Public Health representatives considered a number of partnerships very valuable for their School of Medicine, and thus the institution overall, but of little value to their schools. Many of the higher-value partnerships for Nursing and Public Health were mentioned only by representatives of these programmes, with the general exception being the current or past overall head of the College of Health Science and/or the respective teaching hospital who sometimes also mentioned them.

The value of some partnerships changed over time depending on the level of activities, often in line with external funding. A MU representative perceived the value of the partnership with Maastricht University, Netherlands, decreased to low from high after MHO^10^ funding ended, although all MU representatives who rated this partnership rated it “high value”. A UoN representative stated that over the long-term the partnership with the University of Maryland was medium-value but “at the level of current engagement [i.e. combination of activities and funding] you can actually call it high”. University of Maryland was a partner, along with the University of Washington, in UoN’s Medical Education Partnership Initiative (MEPI) project, PRIME-K, starting in 2010.

For some partnerships, perceptions of value varied significantly between senior representatives within the same HPP. For example, at MUHAS, one representative described the construction of and service provided through care and treatment clinics at health centres in partnership with an USA university and the city council of Dar es Salaam as “… important to MUHAS because we were providing care to people with AIDS, our profile went up since we were involved in the construction of the clinics”. Another representative also rated the partnership as “high-value”, but concluded, “… they [the HIC partner] could have done more”. A third representative rated it “medium-value” because of the high research output but was “very disappointed” there wasn’t more capacity building in research, especially since the USA university was training multiple of its own PhD students directly through the partnership but only supported one Tanzanian PhD student. The same representative contrasted this with the PhD capacity building results Scandinavian partnerships helped MUHAS achieve. Other representatives also lamented the lack of capacity building for MUHAS through the project with the USA university, this time contrasting it to the capacity building outputs gained through partnerships with Norwegian (University of Bergen, especially) and Swedish universities that combine research and PhD obtainment.

The approach of the USA partner mentioned above at MUHAS contrasts with MU’s partnerships with IU and other AMPATH Consortium members. IU has led the partnership with a “lead by care” model that prioritizes healthcare service delivery and includes education, research and infrastructure development too, leading one MU representative to answer if there was an overall objective to the partnership: “Yes, to improve the region. To assist the Ministry of Health in developing a comprehensive care model in western Kenya”.^11^ However, another MU KI credited Linkoping University more for overall support to the College of Health Science for sponsoring Masters and PhDs for faculty and exchanges of nursing students.

## Interpretation of Findings

### General characteristics of Higher-Value Partnerships

All higher-value partnerships shared three general characteristics. One, the outputs and outcomes were a priority need for the representative(s), their School(s), College of Health Science or provided an important service to the community or society, such as responding to the HIV epidemic. The stated mandates of the universities are to provide education, research and service. A partnership can focus on any or all of these components, and at any level; for example, education includes undergraduate or post-graduate work.

Two, the long-term capacity of the focus university to fulfill its mandate was increased. Nuance was expressed by many KIs. Supporting long-term capacity development is fairly clearly realized when faculty members earned their PhDs at a partner university, a plaque is seen on a laboratory, library or ward of a hospital thanking a partner, or reads that a degree programme was started with the support of faculty from a partner university. The Swedish Red Cross University College (SRCUC) was considered to be providing long-term capacity support to KCMUCo although its main support was providing two Nursing students on exchange each semester while sending six Swedish students and faculty mentors to KCMUCo. Although the student exchange ratio was 3:1, SRCUC was a dependable long-term partner in providing the exchanges and securing the funding for them. By maintaining the exchange for over 10 years, year after year, the exchange was *de facto* institutionalized such that it was part of KCMUC’s nursing programme and easy to do, thereby minimizing transaction costs.

Three, the overall capacity building benefits realized by the focus university were perceived to be fair when compared to the benefits realized by the international partner(s). The exchange did not adhere to 1 to 1 reciprocity, but the partnership had to be perceived to be providing sufficient benefits to the focus university such that the international partner isn’t felt to be benefiting significantly more.

### General characteristics of lower- and medium-valued partnerships

#### Insufficient reciprocity

Partnerships with extremely unbalanced representation in activities and, therefore, outputs (e.g. many fewer PhDs earned; student participating in a bi-directional exchange at a ratio of 15 to 1) were considered lower- or medium-value. Imbalances were most commonly observed in partnerships that focused mainly on trainee placements for undergraduate and Masters students. Nineteen (19) partnerships focused principally on trainee exchanges. Twelve (63%) were calculated to be lower-value and the remaining 7 (37%) medium-value. The majority of the direct trainee beneficiaries were trainees from HIC-based universities. In multiple cases, groups of trainees came from European and North American universities to some of the focus countries multiple years in a row without any, or only one, trainee from the East African universities going the other way.

#### Imbalance between southern partners

Three representatives of a focus university identified insufficient reciprocity within one consortia partnership led by a Southern university. They expressed strong opinions about the lack of benefits (PhD students supported by the project) their university received through the partnership. One KI stated, “instead of being considered a colleague we are being seen as a competitor … it should have been our brother university”. A project representative, based at another African university, however, noted that the selection criteria for candidates – strictly merit – was established and agreed to by all parties in advance. The best candidates were selected using a transparent process.

Examples of power imbalances detrimental to the perceived benefits of partnerships were found to exist within both North-South and South-South partnerships. One KI stated that representatives from an African partner university who were supporting the development of an academic programme wrote to them stating they needed to own the outputs of the programme. The focus university representative then stated their university therefore terminated the partnership. A publication, not including the focus university representative as a co-author, tells a different story. This situation is either an example of power imbalance within a partnership or different perspectives of an event. In either case it is another example showing that power dynamics and/or communication are important to consider in South-South partnerships too. In fact, when resources are scarce, it is possible that the politics of resource allocation could be more intense between partners. Discussing partnerships between African universities, another KI at the same focus university concluded, “we are all struggling to develop as it were.”

#### Limited Scale of Participation

Two partnerships with only one representative involved from the international partner university were perceived to be lower- or medium-value. The individual in the medium-value case resided at the focus university for long periods within a three-year period. In the lower-value case, the partner did short placements over a number of years. In both cases, the representatives of the international partners were unable to attract colleagues from their country to participate in the partnership.

CARTA^12^ was mentioned by Schools at both MU and UoN. Each School, Public Health and Nursing, respectively, had one PhD student supported by the Consortium. In addition to the student, CARTA was valued for the mentoring the PhD supervisors. However, the scale of the partnership is limited so capacity will be increased slowly.

### Categorizing Partnership Types

Applying Kernaghan’s five types of partnerships, 121 of 125 (97%), could be classified as either collaborative or operational. We categorized the 4 (3%) outliers as contributory (1), consultative (2) and phoney (1). The one partnership considered to be contributory was stated to be “very high” value by the representative who mentioned it because the contributory partner was able to secure a grant that would be implemented by another international partner and one of the focus universities. The first international partner in question was registered in the country but the second partner wasn’t. This allowed funds to pass through the contributory partner to the international partner that was not registered. Both consultative partnerships were one-time visits to another university by a KI who was a member of a team establishing a new university. The phoney partnership had physicians from a HIC trying to establish a research partnership with a nursing program. However, we think it may be more appropriate and useful to describe this partnership as “neo-colonial” instead of phoney since it is questionable if the international partner was trying to manipulate the East African university representative and neo-colonialism is often discussed in the partnership literature [20]. In this case, it appears that the physicians may have been trying to simply partner with the focus university to pursue their research interests. We also found that certain operational partnerships could be considered neo-colonial if one considers the power imbalances and control of project resources. Some one-way trainee partnerships that only placed HIC students at focus universities could also be considered neo-colonial – when no or very limited tangible benefits are being gained by students or faculty of the focus universities.

In numerous, but not all, of the higher-value partnerships, faculty from the international partner resided in the city of the East African partner university and worked at the focus university. Examples included Indiana University, Duke University and University of Toronto at MU, Ludwig Maximilian University of Munich (LMU) and University of Manitoba at UoN, and London School of Hygiene & Tropical Medicine (LSHTM) and Radboud University at KCMUCo.. There was no example of a Swedish university having long-term residential faculty placements at any of the four institutions, although a total of six Swedish universities at 3 of the 4 focus universities were calculated as being higher-value.

## Discussion

### Many types of partnerships are valued highly

Using Kernaghan’s framework, we found most partnerships to be collaborative or operational, although many clearly mixed the two depending on the activity. Some of the higher-value partnerships had core characteristics of operational partnerships; namely, when decision-making isn’t shared and power largely remains with one partner. The MU-Maastricht partnership is one example where, in the long-run, the durable outputs (the LRC – library, PBL pedagogy, faculty earning PhDs) are what were stressed by representatives.

In general, the literature about international university partnerships puts forward normative guidelines of mutuality, shared resources, and long durations amongst the array of success factors of partnerships [10,31,32,33,34,35,36]. On the surface, much of this partnership literature does not clearly allow for sufficient nuance when providing guidance about how to manage partnerships [10,31,35,36,37] based on the specific context of the partnership. For example, neither efficiency nor, at times, maintaining control are clearly identified as being of fundamental importance, although as Buse and Harmer [33] argue, they could be consistent with local needs or realities at a given time and thus potentially adhering to “best practice” guidelines. Casey [34] mentions the need for balance between power-sharing and control when discussing leadership and managing change.

Other frameworks for examining global health partnerships complement Kernaghan’s; for instance, one presented by Brinkerhoff and Morgan [38] who characterize capacity development activities in terms of: 1) being treated as a project or program; 2) using a strategy of incrementalism, or; 3) being characterized as emergence – an undirected process of collective action. Both partnerships involving German universities started with 10-years of DAAD funding. Heidelberg’s with MUHAS ended after 10 years and was only ever project-based. LMU’s with UoN started as a project and continues. It appears to be an important foundation block for the many ophthalmology activities in East Africa, including the development of ophthalmology programmes at KCMUC and Makerere and the establishment of College of Ophthalmology of Eastern Central and Southern Africa (COECSA) headquartered in Nairobi. Although starting as a project, the collaborative nature of the LMU-UoN partnership was evident from the beginning, as evidenced by the joint-paper titled – *The Role of Traditional Medicine in Ophthalmology in Kenya* [39] – published only five years into it.

It is also useful to consider the utility of The Eight Rungs of Arnstein’s [40] *Ladder of Participation* for examining typologies of global health international partnerships. The eight rungs are divided up into three levels: 1) Lower – non-participation, which consists of manipulation and therapy; 2) Middle – tokenism, which consists of informing, consultation and placation tokenism; and 3) Upper – Decision-Making, which consists of, partnership, delegated power and citizen control. A partnership or a project can commence when a focus university, or programme or school within it, is at various stages of development or maturity. How partners interact will correspond to the experience and knowledge of each representative in the partnership, the level at which each partner university can engage, and the type of partnership it is. While the approach used within a partnership should always be respectful, it may not be appropriate for it to be collaborative at a given stage of an intervention or a specific project.

### Too appreciative of partnership results?

The comprehensiveness of some partnerships is overstated in the literature. For example, Mulvihil and Debas [10] report that one of the success factors of the MU-IU relationships is “collaboration among virtually all major disciplines at both schools”. While it is true that there have been interactions between representatives of Medicine, Nursing and Public Health from the two universities the intensity and scope of the interactions between the three faculties were uneven. Extrapolating the results of one particular component of the programme, for example HIV/AIDS prevention and care [41], to all activities of a partnership and, seemingly the entire College of Health Sciences, as Mulvihil and Debas do, overstates the breadth of partnership benefits for each HPP and all three components (education, research and service) of an academic health sciences centre. This is especially a risk when an Appreciative Inquiry (AI) approach [42] is used by those writing about their partnerships, since the “positivity” of AI’s action-research approach for organizational development is often emphasised by its users instead of its “generativity” [43], resulting in limitations being downplayed [44]. An IU KI comment that they have done the “best job” in care, a “commendable” job in research and were “weakest” in education supports this. The KI continued by stating that the MU-IU MEPI grant was designed to address education weaknesses, but unfortunately the grant wasn’t secured.^13^ In addition, in the case of MU, the contributions of other partners in supporting the development of its College of Health Sciences are also valuable, both those within the AMPATH Consortium – such as Brown University in tuberculosis [45], Duke University in cardiology [46] and the University of Toronto in reproductive health [47],^14^ – and others partnering with MU outside the AMPATH Consortium – such as Linköping (student exchanges, Nursing) and Maastricht Universities (Learning Resource Centre – LRC, problem-based learning – PBL, and PhDs).

By interviewing a range of representatives from the focus universities nuance was gained about many of the partnerships lacking in the literature as some published surveys on global health partnerships seek the perspective of only one representative from an institution in a partnership [48]. Whether the individual is directly involved in the partnerships or from Medicine, Nursing or Public Health will influence what is reported and the overall perspective of the benefit of the partnership. Furthermore, it is likely that nuance is often not reported in published work about partnerships. An interviewee in this study noted that it was decided that they would not report their “dirty laundry” in a peer-reviewed publication about a component of their partnership.^15^

### Perception of value is relative and education needs to remain a priority

Comparing the value of partnerships across disciplines, duration, changing contexts, not to mention the differences in the scale and resources involved in each partnership is not easy. KIs perceived the value of specific partnerships relative to the actual tangible benefits their school, or institution, gained from the partnership and the perceived value of other partnerships in which they were or are involved. Small-scale partnerships of short-durations (e.g. three years) that focused on clear needs of representatives of the focus university were highly-valued. In contrast, there are examples of larger-scale, longer-term partnerships at the same institutions that were not considered higher-value by some representatives because the partnerships were seen to benefit the international partner more. This supports the normative statement by Mulvihill and Debas [10] that successful academic global health partnerships “should be primarily based on the needs and priorities of the less-resourced party” [p. 510].

While many of the global health partnership toolkits focus on research partnerships [31,36,48], partnerships that emphasised education activities including support for pedagogy, post-graduate training, international exposure for undergraduates first were considered to be of more value for strengthening the capacity of the focus universities. A tool introduced here for measuring the relative value of partnerships is the *exchange ratio of trainees*, to keep track of the actual number of trainees involved in partnerships each year and compare the outputs between partners.

### Power dynamics exist within all partnerships: south-south partnerships should not be idealized

There are many examples in the literature of power-imbalances existing within partnerships between HICs and LMICs [20,35,49], but South-South partnerships are not exempt. Several focus university representatives were disappointed with the approach followed by international partners from SSA or the benefits they gained from the South-South partnerships.

In one example, it was perceived that an international partner wanted to continue to own the curriculum once it was established. In another example, it was felt that the benefits of the partnerships were not spread equally, as the lead partner received more trainees. Even if the selection process and terms are agreed to by all parties in advance, if a partner does not feel it is benefiting sufficiently relative to other partners the sense of partnership may be questioned. In both cases, these partnerships linked more established Southern universities with younger universities. A more established Southern partner can appear to dominate a South-South partnership in the same way a more established Northern partner can. There are and will be differences of perspectives among actors and institutions. There are interests at stake among Southern universities just as there are among Northern universities (which are often in direct competition with one another, implicitly and sometimes explicitly) and therefore power and interest dynamics are at play in South-South partnerships just as they are in North-South and North-North partnerships. This is the case even when there are agreed-upon MOUs between parties – such MOUs do not guarantee that interests and perspectives and interpretations will always align. That there continues to be some kind of comforting myth that South-South relationships are necessarily and intrinsically non-competitive and even without any differences of interest or perspective is what is surprising.

### Strengths of partnerships maintaining focus on core objective and coordinating with others

Some KIs reported that the narrowness of partnerships was a weakness. Our findings suggest however that maintaining focus on specific, narrower objectives may be crucial to ensuring that results can be realized and sustained over many years. Indiana University has maintained its focus on supporting the School of Medicine Departments of Internal Medicine, Paediatrics and Surgery while encouraging other universities interested in joining the AMPATH Consortium to lead in supporting MU in other disciplines. Similarly, Karolinska Institute is the overall lead for the Swedish universities partnering with MUHAS and has principally supported the School of Medicine whereas Uppsala has supported Reproductive Health and Umea Nursing and Public Health. In the case of the AMPATH Consortium, the coordination of partners has been done by Indiana University. By working through Indiana University both Duke and Toronto were likely able to partner with MU more quickly and produce results faster than would have been possible without coordinating with Indiana University. Both were considered higher-value partners by MU representatives approximately five years after starting.

### The significance of some lower- and medium-value partnerships should not be minimized

Partnerships determined to be lower- or medium-value should not be considered unimportant. The importance of them is greater than simply future potential. Sometimes they provide opportunities that were stated to be very important to the focus university, although on a limited basis. Consider MUHAS’ partnership with Saint John of God College of Health Sciences in Mzuzu, Malawi. Saint John provided MUHAS’s nursing school placements focusing on mental health without appearing to ask for much, if anything, in return.

## Conclusions

One-quarter of global health partnerships at four East African universities are considered higher-value by their representatives for building their HPPs’ capacity. The partners come from within Africa, Europe and North America. In some cases, the perspectives of the same partnership vary significantly among representatives. Overall, representatives of the focus universities placed greatest value on partnerships that supported: post-graduate training, especially, PhDs; support of new pedagogy and disciplines; infrastructure development; and, international learning experiences for their students. Collaborative partnerships may be the ideal type of partnership in theory, but sometimes an operational, contributory or consultative partnership may be as or more appropriate within a given context. A collaborative approach may not be justified for all activities or in certain contexts, although as capacity increases at an institution this is less likely. Overall, international partners who prioritize the needs of the focus university, support it in increasing its long-term capacity and best ensure the capacity benefits realized favour the focus university will be valued most. Representatives of universities interested in forming new partnerships should explore coordinating with existing partners or filling gaps in past partnerships to achieve higher-value status more quickly. There are administration and transaction costs associated with coordination but the inefficiency of not coordinating partnerships should be considered too. Ultimately, the role of coordinating global health university partnerships at each university rests with each university. International partners and donors should support the coordination efforts of LMIC universities.

## Additional Files

The additional files for this article can be found as follows:

10.29024/aogh.20.s1 Appendix 1. Detailed findings of Higher-Valued Partners of each Focus University.

10.29024/aogh.20.s1 Appendix 2. Table of Partners by Country and Value of Partnership.

10.29024/aogh.20.s1 Appendix 3. Table of Higher-Value Consortia – coordinating and partnering universities.

10.29024/aogh.20.s1 Appendix 4. Interview Guide for Phase 2 – FGDs with Senior Lecturers and Lecturers.

10.29024/aogh.20.s1 Appendix 5. FGD Guide for Phase 2 – Students.

10.29024/aogh.20.s1 Appendix 6. Generic Interview Questions for International Partners – Phase 3.

## Footnotes

Notably, the second paragraph of the section – Invest in People, Institutions, and Capacity Building – of the IOM report begins. “Although there has been little rigorous evaluation to parse the most promising aspects of the institutional partnership model…” [4, p. 113–117].

Vinkers et al. [16] found that the use of positives words in publications increased by 880% between 1974 and 2014s. They found that the use of the word *innovative*, specifically, increased by over 2000%. However, it should be noted they found that the frequency rate of positive and negative words in titles and abstracts by authors affiliated with four English-speaking countries declined during the last 10 years of the research period.

These included current and immediate-past deans of medicine, nursing, public health and heads of the college or university (i.e. vice-chancellors, provosts and principals.

129 partnerships were identified at the four focus universities. Three consortia were mentioned by at least two of the universities. Thus, there were 125 distinct partnerships.

As noted previously, 129 partnerships were identified at the four focus universities. Three consortia were mentioned by at least two of the universities. Thus, there were 125 distinct partnerships.

Trainees at some level participated from all three disciplines at all four universities, except for public health trainees at KCMUCo.

When partners of Public Health and Nursing programs were considered *high value* by the senior representative respondents of these schools but not the other senior representative(s) of the focus university these partners were classified as *higher-value* too, unless another representative(s) of the faculty or university stated strongly why the partnership should not be considered high value.

The World Bank’s 2014 classification of countries by income group was used. www.worldbank.org.

Kenya was classified by the World Bank as a low-income country when the data was collected.

MHO stands for Medefinansieringsprogamme voor Hoger Onderwyssamewerking (Dutch). In English it means the Joint Financing Programme for Higher Education Co-operation [30].

See: http://www.ampathkenya.org/our-model (Accessed 16 February 2017).

CARTA is the Consortium for Advanced Research & Training in Africa. It was not determined to be a higher-value partnership by this study because at most 1 representative at a focus university mentioned it. This is likely due to its scale. The structure and processes used by CARTA appear to be respected by its participants and members however.

This is somewhat ironic since Frenk, J, L. Chen, et al. [6] identify the MU-AMPATH Consortium model, led by IU, as one of the partnership models that “sparked” the launch of MEPI.

The fields appearing in parenthesis were stated by KIs of MU. Examples of corresponding publications are presented. MU representatives emphasised Internal Medicine, Paediatrics and Surgery as the Departments where Indiana University supported capacity building in the College of Health Sciences the most, in addition to HIV/AIDS prevention and treatment and establishment and support of RSPO. It is important to note that the activities of the universities in the AMPATH Consortium are not limited to the Department that they support. Representatives from Brown, Duke and Indiana have all been involved in Reproductive Health activities and while MU representatives identified Toronto as the lead North American university Indiana University has been a co-lead within the Consortium and has had long-term faculty placements in Eldoret.

The on-line Merriam-Webster dictionary defines *dirty laundry* as, “The private matters whose public exposure brings distress and embarrassment – called also *“dirty linen”* (*www.merriam-webster.com, Accessed 13 February 2017*). However, it appears likely that significant challenges, not only *private matters*, partnerships experience are unlikely to be reported in publications.
