# Supplementary material for: What Makes International Global Health University Partnerships Higher-Value? An Examination of Partnership Types and Activities Favoured at Four East African Universities
Source: Ann Glob Health. 2018 Apr 30;84(1):139–50. doi: 10.29024/aogh.20 (PMC6748237; doi:10.29024/aogh.20)
Supplement: Appendix 1. — Detailed findings of Higher-Valued Partners of each Focus University. [file agh-84-1-20-s1.pdf]

Appendix 1: Detailed findings of Higher-Valued Partners of each Focus University

A1.1 Moi University

Table A1: MU's Higher-Value Partnerships, listed in order of most mentioned by senior representatives

#	Name of Institution	Country	Years Active	Currently Active	HPPs Involved[i]	AHSC Comps Involved	Identified by X Reps (n=10)	Strengths Mentioned by Focus University KIs	Limitations Mentioned
1	Indiana University	USA	23	Yes	Med & Nur	All	10	Service: internal medicine; paediatrics; surgery; Education: faculty & student exchanges; Research: clinical trials; development of RSPO; Infrastructure: Mother-Baby Hospital	Support of Schools of Nursing and Public Health
2	Linköping University	Sweden	23	Yes	Med & Nur	Edu & Res	8	PhDs & Master's; Problem-Based Learning; Student Exchanges	Approach to PBL different to MU's
3	Brown University	USA	16	Yes	Med & PH	All	5	TB service (hospital & community), education and research; education exchanges	Limited in personnel
4	Maastricht University	Netherlands	23	Yes	All	Edu & Res	5	Infrastructure: LRC; Problem-based learning; PhDs	Did not support project management support at MU when building LRC
5	University of Toronto	Canada	5	Yes	Med & PH	All	5	Reproductive Health (hospital & community); exchanges; Public Health	Too narrow: mainly Reproductive

[i] Involvement does not denote higher-value for each HPP mentioned. In many cases, more than one HPP was involved but representatives of only one or two of the schools considered the partnership high-value for their school.

									Health
6	Duke University	USA	4	Yes	Med & PH	All	4	Cardiology: service; education; research	Too narrow: mainly Cardiology
7	McMaster University	Canada	4	No	All	Edu	2	Problem-based learning, including planning workshops	
8	One Health Central and Eastern Africa (OHCEA)	Consortium	3	Yes	PH	All	2	Exposing faculty & students to issues of human, animal & environmental health; on-line PBL with Tufts	

MU's partnership with Indiana University was mentioned by every representative in Phase 1. Although it was stated to be the most important partnership to the College of Health Science by almost all representatives, some Nursing and Public Health representatives didn't list it as a significant partner for their School or stated its direct capacity building support was limited for their School even though several their faculty members were involved in the AMPATH program that MU and Indiana representatives implement. One Nursing representative regarded the equally long-standing partnership with Linköping University to be of greater value to their School. The partnership with Indiana was stated to be building capacity in many areas including service, research, education, infrastructure and support services such as the Research Services and Projects Office (RSPO). One Phase 1 representative compared the manner in which Maastricht University and Indiana built capacity. When the former established the Learning Resource Centre (LRC) at MU they based an individual to reside in Eldoret for the duration of the multi-year project; stating, "… this was total control of the work, as opposed to the way AMPATH (i.e. IU) built RSPO". However, a Maastricht University KI noted that they had a MU counterpart. Three other members of the AMPATH Consortium, a consortium of North American universities led by Indiana University, were identified as higher-value partnerships by MU: Brown University; Duke University; and, University of Toronto.

MU KIs identified McMaster University although it hadn’t formally partnered with MU for over 10 years. McMaster is credited for being instrumental in assisting MU’s HPPs in establishing its problem-based learning curriculum (PBL). Maastricht University, Linköping University and Ben-Gurion University of the Negev in Israel were also identified for their support of PBL at MU.

A1.2 University of Nairobi

Table A2: UoN’s Higher-Value Partnerships, listed in order of most mentioned by senior representatives									
**#**	**Name of Institution**	**Country**	**Years**	**Active**	**HPPs Involved**	**AHSC Comps**	**Identified by X Reps (n=9)**	**Strengths Mentioned by Focus University KIs**	**Limitations Mentioned**
1	University of Manitoba	Canada	35	Yes	All	All	8	Infrastructure: UNITAD; Research: HIV/AIDS Research; PhDs	
2	University of Washington	USA	25	Yes	All	All	8	Education thru MEPI, especially rural retention of physicians; PhDs and Master's; Research: mentorship & support	Nursing
3	University of Maryland	USA	25	Yes	All	Edu & Res	7	Education: HIV/AIDS (PACE); ID Fellowship; mentorship	
4	Ludwig Maximilian University of Munich (LMU)	Germany	30	Yes	Med	Edu	3	M.Med Ophthalmology; sub-specialty support; equipment	
5	One Health Central and Eastern Africa (OHCEA)	Consortium	3	Yes	PH	All	2	Curriculum development; faculty exchange visits; Leadership training	

Four of the five UoN partnerships determined to be higher-value for building the capacity of its HPPs were at least 25 years old. University of Manitoba is the College’s oldest and stood out for securing funding for the construction of UoN’s Institute of Tropical and Infective Diseases and PhD training. Although initially focused in Medicine and Public Health, links had been established with the School of Nursing recently. The University of Washington and Maryland activities have also historically been focused on Medicine and Public Health, although through PRIME-K partnerships activities have reached Nursing too. However, Nursing had no higher-value partnerships. Public Health’s only higher-value partnership was OHCEA.

The partnership with Ludwig Maximilian University of Munich and its Eye Hospital, operating strictly with Medicine established the M.Med. in Ophthalmology, was stated to be high-value by the three representatives who mentioned it although none of them were involved in it directly. Initial funding (10 years) was provided by DAAD – the German Academic Exchange Service – in 1978 to establish the degree program.[ii] The first student graduated in 1980. He was Kenyan, as were the next four. The first foreign student graduated in 1984. By 2013, 167 students had graduated, 99 (59%) Kenyan and 68 (41%) foreigners. Fifty-seven (84%) of the foreign graduates were from 16 countries from the WHO Africa Region, 5 Eastern Mediterranean Region, 4 European Region and 2 South-East Asia Region. As a UoN Phase 2 representative concluded, “Through University of Munich they negotiate for funding, physical facility development, they also participate in training, they source and they get equipment for student ophthalmologists and through the University of Nairobi they have funded the University of Nairobi to train most of the ophthalmologists in sub-Saharan Africa”.

[ii] DAAD’s initial funding was for a 10-year project. The majority of the funding went to cover the cost of topping up the salaries of the German participants. A ten-year MOU was signed between the University of Nairobi and LMU’s Eye Hospital. Two subsequent MOUs were signed. The fourth and current MOU was signed in 2014.

A1.3 Kilimanjaro Christian Medical University College

Table A3: KCMUC's Higher-Value Partnerships, listed in order of most mentioned by senior representatives

#	Name of Institution	Country	Years	Active	HPPs Involved	AHSC Comps	Identified by X Reps (n=12)	Strengths Mentioned by Focus University KIs	Limitations Mentioned
**1**	Duke University	USA	16	Yes	All	All	12	Education thru MEPI, especially research grants and ICT; Research: HIV & Malaria	Was mainly research before MEPI
**2**	Radboud University Medical Centre	Netherlands	13	Yes	Med	Edu & Res	10	PhD & Master's; Infrastructure: KCRI building; Research	Mainly Medicine
**3**	University of Copenhagen	Denmark	12	Yes	Med & PH	Edu & Res	9	General Education and Research capacity building	Mainly Medicine
**4**	London School of Hygiene & Tropical Medicine	UK	12	Yes	Med & PH	Edu & Res	8	Epidemiology Lecturers; PhDs & Master's; Research & Research capacity building;	
**5**	Karolinska University Hospital	Sweden	13	Yes	Nur	Edu & Res	5	Student exchangs; KCMUC lecture in Sweden	
**6**	Red Cross University College	Sweden	14	Yes	Nur	Edu	5	Student exchanges (ratio 1:3); mentoring of academic staff	

All KCMUCo representatives identified Duke University as a significant partner as a result of the size and scope of their MEPI grant.[iii] The opportunity for all KCMUCo faculty to compete for small research grants through MEPI was one example how this project's capacity building reach extended beyond Medicine.[iv] However, Radboud University Medical Centre in Nijmegen was mentioned by one representative as the most valuable partnership because of its support of Masters and PhD obtainment by faculty in Medicine. LSHTM's support of KCMUCo was perceived to be greatest for Public Health in education and research and for research with the research centre, Kilimanjaro Christian Research Institute (KCRI). One senior representative considered the partnership high-value for KCRI but low for the College.

A1.4 Muhimbili University of Health and Allied Sciences

Table 6: MUHAS's Higher-Value Partnerships, listed in order of most mentioned by senior representatives

#	Name of Institution	Country	Years	Active	HPPs Involved	AHSC Comps	Identified by X Reps (n=11)	Strengths Mentioned by Focus University KIs	Limitations Mentioned
**1**	Karolinska Institute	Sweden	27	Yes	All	Edu & Res	9	PhDs for faculty; HIV Research; Support Quality Control Laboratory	
**2**	University of Bergen	Norway	25	Yes	All	Edu & Res	9	PhDs and Master's; Research within PhDs	
**3**	University of California at San Francisco (UCSF)	USA	8	Yes	All	Edu & Res	9	Competency-Based Learning Pedagogy thru Academic Learning Project (ALP); Infrastructure; Research	

[iii] The value of KCMUCo's MEPI grant, like all MEPI grants, was approximately US$10,000,000 over 5-years.
[iv] There may have been other examples, but the small grant opportunities were the ones emphasised by KIs.

**4**	Dartmouth College	USA	23	Yes	Med & PH	Edu & Res	9	Research; 2-way exchanges; support est. Centre for HIV/AIDS; mainly School of Medicine (Internal & Microbiology)	
**5**	Uppsala University	Sweden	26	Yes	All	Edu & Res	8	PhDs & Master's; Infrastructure Development - Laboratory	
**6**	Umea University	Sweden	26	Yes	All	Edu & Res	7	PhDs & MSc; Research within PhDs; 2-way Trainee Exchanges	
**7**	Makerere University	Uganda	25	Yes	All	Edu	4	Leadership Education; MScNursing-Midwifery	
**8**	University of KwaZulu-Natal (UKZN)	South Africa	5	Yes	Nur & PH	Edu & Res	4	M.ScNursing-Mental Health; Public Health Policy Research	
**9**	Dalhousie University	Canada	5	No	Med & Nur	Edu	4	Supported establishment of BScNursing	
**10**	NOMA (Norad's Programme for Master Studies)	Consortium	5	Yes	Nur	Edu	3	Establishment MScNursing; regional network with Ethiopia, Kenya & Uganda	
**11**	University of Nairobi	Kenya	25	Yes	All	Edu & Res	3	Clinical attachments (Nursing); Research; External Examiners	
**12**	Boston University	USA	3	Yes	PH	Edu	2	Curriculum Development; 2-way Faculty Exchange	
**13**	University of Heidelberg	Germany	10	No	PH	Edu	2	Establishment of 1-Year MPH over 10-year project.	

All of MUHAS' higher-value partnerships had very clear education foci. The Swedish and Norwegian universities and Dartmouth College were mentioned for training Masters and PhDs. Dalhousie University supported MUHAS in establishing its Bachelor's in

Nursing. UKZN helped Nursing establish a Master's in Mental Health and continued to be external examiners of the graduating students. The partnership with UKZN was one of three South-South partnerships calculated to be of higher-value at MUHAS, the only focus university with higher-value South-South partnerships, although some KIs did identify some South-South partnerships as high-value. University of Heidelberg helped MUHAS' SOPH establish a 1-Year MPH programme. UCSF partnered with MUHAS on a project to transform the entire university's curriculum to being competency-based when it was a priority need for the university because of changing government policy. The Bill and Melinda Gates Foundation provided a multi-million dollar grant for it. A subsequent ~ US$400,000 grant from the Centre for Disease Control and Prevention (CDC) in the USA allowed Boston University to support MUHAS' SOPH in fine-tuning its new competency-base curriculum soon after the project with UCSF ended.

A decanal representative stated why an education partnership that created a degree course was high-value:

> "High, because then we had specialized staff …. Because if you speak from our perspective, if you want to run a university you need to have highly qualified people. But how do you get highly qualified people when the institution itself has a shortage? You need to bring in people from outside to train others here and get their Masters and PhDs. Or, you need to send people to other universities and then when (you) translate that to how much of it is contributing, then you say it's high…. If you train people in specialities it makes more sense, then you can be independent".

A1.5 Higher-Value Partners of Consortia

Table 7: Higher-Value Consortia Partnerships Identified by Senior Representatives of the Four Focus Universities.

Focus Universities	Name of Consortium	HPPs Involved	Country(ies) of Lead(s)	Membership North-South
Moi, MUHAS & UoN	OHCEA	PH (and Veterinary)	Uganda[v]	North-South
MUHAS	NOMA[vi]	Nur	Norway and Tanzania	North-South

[v] Makerere University was the hub, although University of Minnesota was the overall PI.
[vi] See Leshabari S, ChaloNabirye R, Mukamana D, Mill J, et al.[50] for Information about this partnership

Two of the 10 consortia were determined to be higher-value.[vii] One of them, OHCEA – One Health Central and Eastern Africa,[viii] is a network, arising out of a USAID One Health project. It links seven schools of public health (SOPH) and seven veterinary institutions from six countries in central and east Africa. Two USA universities (University of Minnesota and Tufts University) are ex-officio members. OHCEA was the idea of HEALTH Alliance, a consortium of seven SOPH in DRC, Ethiopia, Rwanda, Tanzania, Uganda and Kenya. HEALTH Alliance originated from LIPHEA, another project funded by USAID. The Kenya representatives rated OHCEA high-value but the Tanzanian representatives rated it medium-value. A MU representative commented on the importance of the research, education (PBL with Tufts) and staff exchanges before stating, "I think it will never end. As a network you can lobby for funds from all kinds of placed". Another MU representative stated it was valuable because of the issues involved: building and strengthen capacity to combat the emerging threats from zoonotic diseases. A MUHAS representative who considered the partnership medium-value because such projects are very active "when money is there" but "they do not have a lot of sustainability … and I think this is dangerous". The KI did add that some aspects of OHCEA, like LIPHEA before it, were institutionalized into the curriculum. Another MUHAS representative rated it medium-value, but said it had the potential to be high.

[vii] Note: neither AMPATH nor the Swedish universities partnered with MUHAS were considered consortia for this study since KIs typically mentioned individual universities. For details see Yarmoshuk AN, Guantai AN, Mwangu M, Cole DC, Zarowsky C.[17]

[viii] A Summary of OHCEA's Ten-Year Strategic Plan, March 2011 – 21 can be found at ohcea.org (accessed 21 February 2017).

**Appendix 2: Table of Partners by Country and Value of Partnership**					
**Country of Partner**	**Higher**	**Medium**	**Lower**	**TOTAL**	**% of All Partnerships Higher-Value**
Australia	0	0	2	2	0
Belgium	0	0	2	2	0
Canada	4	0	2	6	67
Consortium	2	5	3	10	20
Denmark	1	1	0	2	50
Egypt	0	1	1	2	0
Germany	2	0	0	2	100
India	0	1	0	1	0
Israel	0	1	1	2	0
Japan	0	0	4	4	0
Kenya	1	1	0	2	50
Malawi	0	1	1	2	0
Netherlands	2	1	1	4	50
Nigeria	0	0	1	1	0
Norway	1	3	3	7	14
Singapore	0	0	1	1	0
South Africa	1	4	3	8	13
South Korea	0	2	0	2	0
Spain	0	1	1	2	0
Sudan	0	0	1	1	0
Sweden	6	1	1	8	75
Uganda	1	1	0	2	50
UK	1	4	6	11	9
USA	9	13	19	41	22
**Total**	**31**	**41**	**53**	**125**	

Appendix 3: Table of Higher-Value Consortia – coordinating and partnering universities			
**Name of Consortium**	**Coordinating University(ies)**	**Country**	**Partners**
Norwegian Program for Master Studies (NOMA) – Regional Masters in Nursing Initiative	Bergen University College; MUHAS	Norway; Tanzania	**Southern Partners:** Addis Ababa University (Ethiopia), Muhimbili University of Health and Allied Sciences (Tanzania), Makerere University (Uganda). **Northern Partner:** Bergen University College (Norway)
One Health Central and Eastern Africa (OHCEA)	Makerere University	Uganda	***African Partners:*** University of Kinshasa School of Public Health (DRC), Faculty of Veterinary Medicine University of Lubumbashi (DRC), Jimma University College of Public Health Medical Sciences (Ethiopia), Jimma University College of Agriculture and Veterinary Medicine (Ethiopia), School of Veterinary Medicine, College of Veterinary Medicine Mekelle University (Ethiopia), University of Nairobi School of Public Health (Kenya), Moi University School of Public Health (Kenya), University of Nairobi Faculty of Veterinary Medicine (Kenya), National University of Rwanda School of Public Health (Rwanda), Umutara Polytechnic Faculty of Veterinary Medicine (Rwanda), Muhimbili University of Health and Allied Sciences School of Public Health and Social Sciences (Tanzania), Sokoine University of Agriculture Faculty of Veterinary Medicine (Tanzania), Makerere University School of Public Health and Makerere University College of Veterinary Medicine (Uganda) ***Northern University Partners:*** Tufts University, USA; University of Minnesota, USA

Appendix 4: Interview Guide for Phase 2 – FGDs with Senior Lecturers and Lecturers

Overall Question: What in your opinion have been or are the ten most important international partnerships since 1991 for strengthening your School to produce health professionals from your country? Please answer the following questions for up to 10 partnerships.

a) Please review the list of international partnerships your institution has on the attached sheet. (List to be presented at the start of the FGD).

b) Are there any international partnerships that you feel have been significant to building the capacity of your institution that are not included in the list? If so, what are they and what did they focus on.

c) Identify key benefits of each partnership from your perspective.

d) Identify key challenges of each partnership from your perspective.

Appendix 5: FGD Guide for Phase 2 – Students

1. Please introduce yourself, state where you are from and why you chose your program and institution of study.

2. What international exchange did you do? When and where? What was the structure of it?

3. Where were the benefits and challenges to you of your international placement?

4. How will what you learned during your international placement help you here?

5. Have you had to do a presentation about your experience?

6. Any resentment from your fellow students who did not go on international placements?

7. What international partnerships do you know about that your institution is involved?

8. What involvement do you have with representatives from international partners here at your home institution?

9. Do you think having participated in an international placement may encourage you to seek international work after graduating?

Appendix 6: Generic Interview Questions for International Partners – Phase 3

Topic: The role of international partnerships in building the capacity of health professional programs in Kenyan and Tanzanian universities.

Keeping information you don't wish shared confidential:
As this study includes a small number of select representatives at a small number or select institutions, it will be difficult to keep certain information from being attributable to you should it be presented. To protect your interests, you will be allowed to review my manuscript before it is completed and submitted or published. I will agree to re-word my writing to better ensure your confidentiality and/or anonymity while not modifying my conclusions if I believe they are valid. You will be given the opportunity to refute my conclusions and I will include them either as a footnote or an appendix in my dissertation.

1. When did you explore partnering with the Kenyan/Tanzanian university?
2. What types of GH opportunities were you interested in establishing? Why?
3. Who else at your university was/is interested in partnering with the host university – faculty and/or students?
4. What have you done with the host university?
5. What has been accomplished in terms of outputs?
6. What were the benefits for the host university?
7. What were the challenges of collaborating with the host university?
8. How did your university benefit from the collaboration?
9. Do you see the partnership ending?

References

50. Leshabarim S, ChaloNabirye R, Mukamana D and Mill J. Looking forward to the East African Countries' Collaboration in Nursing and Midwifery Education, Practice and Legislation. Rwanda Journal. 2015; 2(2): 69. DOI: https://doi.org/10.4314/rj.v2i2.12F
